# DHODH and cancer: promising prospects to be explored

**DOI:** 10.1186/s40170-021-00250-z

**Published:** 2021-05-10

**Authors:** Yue Zhou, Lei Tao, Xia Zhou, Zeping Zuo, Jin Gong, Xiaocong Liu, Yang Zhou, Chunqi Liu, Na Sang, Huan Liu, Jiao Zou, Kun Gou, Xiaowei Yang, Yinglan Zhao

**Affiliations:** 1 State Key Laboratory of Biotherapy and Cancer Center, West China Hospital, Sichuan University, and Collaborative Innovation Center for Biotherapy, Chengdu, 610041 China; 2 The Laboratory of Anesthesiology and Critical Care Medicine, Translational Neuroscience Center, State Key Laboratory of Biotherapy, West China Hospital, Sichuan University, Chengdu, 610041 Sichuan China; 3 West China School of Pharmacy, Sichuan University, Chengdu, 610041 China

**Keywords:** Cancer metabolism, Dihydroorotate dehydrogenase, DHODH inhibitors, De novo pyrimidine biosynthesis, Mitochondria

## Abstract

Human dihydroorotate dehydrogenase (DHODH) is a flavin-dependent mitochondrial enzyme catalyzing the fourth step in the de novo pyrimidine synthesis pathway. It is originally a target for the treatment of the non-neoplastic diseases involving in rheumatoid arthritis and multiple sclerosis, and is re-emerging as a validated therapeutic target for cancer therapy. In this review, we mainly unravel the biological function of DHODH in tumor progression, including its crucial role in de novo pyrimidine synthesis and mitochondrial respiratory chain in cancer cells. Moreover, various DHODH inhibitors developing in the past decades are also been displayed, and the specific mechanism between DHODH and its additional effects are illustrated. Collectively, we detailly discuss the association between DHODH and tumors in recent years here, and believe it will provide significant evidences and potential strategies for utilizing DHODH as a potential target in preclinical and clinical cancer therapies.

## Introduction

Cellular metabolism is the basis of all biological activities. Metabolic dysregulation, an emerging hallmark of cancer, contributes to maintain cell proliferation, migration, and differentiation during tumorigenesis and progression [1]. The metabolic enzymes play a vital role in this process and become important targets for anti-cancer drug development [1].

Pyrimidine nucleotides play a significant role in tumor cell proliferation as precursors of RNA and DNA [2]. There are two ways for the synthesis of pyrimidine: the salvage synthesis pathway and the de novo synthesis pathway [2]. In resting or fully differentiated cells, pyrimidines are mainly provided by the former. While in highly proliferative cells like tumor cells, the latter is usually highly active to meet the increased demand for nucleic acid precursors and other cellular components [2]. Compared with normal proliferous cells, there is a significant imbalance of pyrimidine metabolism in cancer cells which is stringently linked with tumor transformation and progression [3].

Dihydroorotate dehydrogenase (DHODH), located in the inner membrane of mitochondria, is an iron-containing flavin-dependent enzyme and plays a crucial role in the de novo synthesis of pyrimidine [4]. DHODH catalyzes the conversion of dihydroorotate to orotate in a redox reaction, which is the fourth of six universally conserved enzymatic reactions in the pyrimidine de novo synthetic pathway [5]. The essential role of DHODH in pyrimidine synthesis and mitochondrial functions attracted much attention during past decades [6]. In 1959, its role in tumor progression was first reported, and increasing evidences indicated that DHODH expression and activity are directly related to cancer progression. Therefore, extensive efforts have been made to develop DHODH inhibitors for cancer treatment. The functional or biochemical role of DHODH in cancers or other diseases’ development as well as of their related inhibitors has been well reviewed in several papers [7–9]. However, it remains a hot topic and has been further investigated for the detailed mechanisms of this metabolic enzyme in multiple malignancies or other diseases. Therefore, due to the quick updates of some novel knowledge of this protein, especially in its inhibitor development, it is necessary to recap some new findings.

This review aims at providing an overview of recent findings in DHODH biology and inhibitor development. The relationship of DHODH with de novo pyrimidine metabolism and mitochondria system in various cancers is discussed. The broad-ranging progresses in recent years made in DHODH inhibitor development for cancer therapy are summarized.

## Characteristic property of DHODH

*DHODH* gene, located in the open reading frame (ORF) of human chromosome 16q22 with full length of 1191 bp, encodes DHODH protein with 397 amino acid sequences [4]. The crystalline enzyme was deemed to contain flavin mononucleotide (FMN), flavin adenine dinucleotide (FAD), and iron [10].

According to sequence similarity and subcellular location, DHODH is divided into Class 1 and Class 2 DHODHs. Soluble class 1 DHODHs are further classified into class 1A, class 1B, and class 1S, which are all located in cytoplasm. Class 1A are homodimeric proteins and found in Gram-positive bacteria. Class 1B DHODHs is a dimer of heterodimers and usually found in prevalent in Gram-positive bacteria, consisting of two distinct proteins. The S DHODH is a newly found type which is incapable of utilizing any of the natural electron acceptors. It uses serine as catalytic base, which is special for a cytosolic DHODH [5, 11]. Class 2 DHODHs are monomeric proteins that attach to the mitochondrial inner membrane in eukaryotes and some prokaryotes [11–14]. Class 1A and class 1B share approximately 30% sequence identity, whereas soluble class 1 and membrane-bound class 2 DHODHs share approximately 20% of sequence identity [11].

DHODH was first isolated in 1953 from extracts of *Clostridium oroticum* (CoDHODH) [11, 15]. The first crystal structure of flavin containing DHODH has been determined from *Lactococcus lactis* in 1997 (LlDHODH) [16], and the structure of human DHODH in complex with anti-proliferative agents was solved in 2000. Structural studies show that DHODH contains two domains, a large C-terminal domain and a smaller N-terminal domain, which are attached by an extended loop. The N-terminal extends approximately 40 residues folds into two α-helices (αA and αB) linked by a short loop and related to membrane association [11]. The cytosolic larger C-terminal contains the redox site, the N-terminal domain forms a tunnel inside the membrane that hides the FMN binding site [2, 9, 17]. Notably, the majority of the structural elements and residues relevant to both FMN and substrate binding are conserved in classe1 and 2 of DHODHs [11]. Ubiquinone, utilizing the tunnel to approach the FMN cofactor for participating in the redox reaction, can easily diffuse into the mitochondrial inner membrane [18, 19]. Ubiquinone Q_10_ binds the signal peptide located in the N-terminus of DHODH, which covers for mitochondrial import and is a transmembrane domain with a microdomain interacting with the mitochondrial inner membrane [20, 21]_._

## DHODH regulates cancer progression

### The de novo pyrimidine metabolism and DHODH

**Fig. 1:**
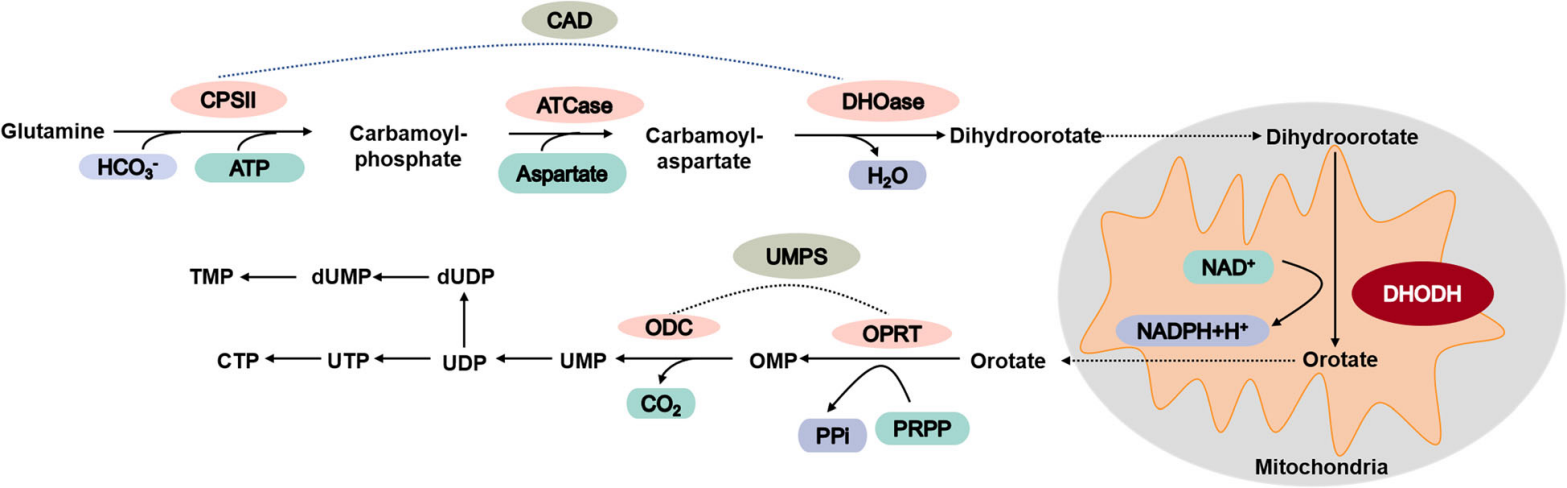
Pyrimidine de novo biosynthesis pathway

Pyrimidines are necessary for the biosynthesis of DNA, RNA, glycoproteins and phospholipids [9]. Pyrimidine nucleotides are synthesized through two pathways: the de novo synthesis pathway and the salvage pathway [22]. Pyrimidines are synthesized de novo from simple precursors with six steps (Fig. 1). The enzymes that catalyze uridine monophosphate (UMP) synthesis include carbamoylphosphate synthetase II (CPSII), aspartate transcarbamoylase (ATCase), dihydroorotase (DHOase), DHODH, and uridine monophosphate synthase (UMPS) [23]. Firstly, glutamine (Gln), ATP and HCO_3_− collectively form carbamoyl phosphate, which is catalyzed by CPSII, a vital enzyme located in the cytosol. ATCase contributes to the formation of the carbamoyl-aspartate following, the pyrimidine ring further is cyclized by DHOase to produce dihydroorotate. Secondly, the flavoenzyme DHODH converts dihydroorotate to orotate which takes place in mitochondria. Orotate further converts to UMP by the UMP synthase UMPS. UMP, a precursor of other pyrimidines and various biological processes, then converts to uridine triphosphate (UTP) by phosphorylation. UTP is subsequently converted to cytidine triphosphate (CTP) with the donor glutamine by CTP synthetase. Meanwhile, the uridine diphosphate (UDP) transforms to deoxyuridine diphosphate (dUDP) by ribonucleotide reductase, with its ribose moiety reducing to deoxyribose. Deoxyuridine monophosphate (dUMP) methylation generates deoxythymine nucleotides (dTMP or TMP) for DNA synthesis [2, 23–25]. In this process, DHODH catalyzes the fourth step in the de novo biosynthesis of pyrimidine by converting dihydroorotate into orotate in a redox reaction in the mitochondria with ubiquinone (CoQ) converting to ubiquinol (CoQH2), which is a substrate of respiratory complex III [2, 11, 26].

As DHODH plays a crucial part in pyrimidine synthesis, it exerts varying effects on different cell types and developmental stages owing to the relative contribution of de novo pyrimidine synthesis to maintaining proliferation. The de novo pyrimidine synthesis has gained a prominent position to satisfy increasing demand for nucleic acid precursors in rapidly proliferative cells such as activated T cells due to the rapidly increased demand for DNA replication and nucleic acid biosynthesis [9, 23, 27, 28]. While in resting or fully differentiated cells, they mainly obtain pyrimidines through the salvage pathway for proliferation [9, 28–31]. Thus, the effect of DHODH is more predominate in rapidly proliferating cells like cancer cells, which may be highly sensitive to inhibition of nucleotide synthesis [32–35].

Consistent with above observations, DHODH blockade by inhibitors or RNA interference exhibited anti-proliferation effect by pyrimidine depletion. Studies manifest that DHODH inhibitors have more potential to treat malignancies which are more dependent on de novo pyrimidine synthesis and have lower pyrimidine salvage activity. For instance, in PTEN-mutant cells which depend upon glutamine flux through the de novo pyrimidine synthesis pathway [29], inhibition of DHODH causes stalled forks due to inadequate nucleotide pools required to support replication. Sustained treatment with DHODH inhibitor further leads to Rad3-related kinase (ATR) activation, leading to a buildup of DNA damage and cell death [29]. Cancer cells are hypersensitive to DHODH inhibitors under the tumor hypoxia and nutrient-deprived microenvironment [27, 36]. Further, depletion of the pyrimidine nucleotide pool, especially UTP, resulting from DHODH inhibition, could impair biogenesis of ribosomes, which activates the tumor suppressor p53 pathway leading to cell cycle arrest [37, 38]. Moreover, DHODH is regulated by several critical transcription factors [24]. The predicted and known regulators of DHODH include E1A-binding protein p300, POU domain, class 3, transcription factor 2 (POU3F2), GATA-binding factor 2 (GATA-2), nuclear factor kappa-light-chain-enhancer of activated B cells 1 (NF-κB1), and proto-oncogene MYC [24]. It might be significant to inhibit DHODH through interfere with these regulatory factors, which can further contribute to pyrimidine depletion and induce death of tumor cells.

Apart from the direct blockade of pyrimidine synthesis, DHODH also exerts additional effects including O-linked *N*-acetylglucosaminylation (O-GlcNAc) [39], senescence [7], AND mRNA translation [40] indirectly by pyrimidine depletion. UMP, the downstream product of DHODH, is a precursor for components required for the assembly of various cellular macromolecules, including phospholipids, glycogen, hyaluronic acid, and proteoglycans, as well as for certain post-translational protein modifications [30, 41], which may account for the above extensive influences of DHODH. For example, O-GlcNAc, a post-translational modification of proteins, can add *N*-acetylglucosamine (GlcNAc) on UDP [42]. UMP reduction by DHODH suppression may result in integral decreases in protein *N*-acetyl glycosylation in acute myeloid leukemia (AML) [43], and reduction of UMP-GlcNAc promotes myeloid differentiation [43]. Given that O-GlcNAc plays a crucial role in myeloid differentiation [44–46], it may explain the mechanism that DHODH inhibition promotes the myeloid differentiation in AML to some extent. Collectively, DHODH inhibition decreases the de novo pyrimidine synthesis and affects many biological processes.

### Mitochondria function and DHODH

**Fig. 2:**
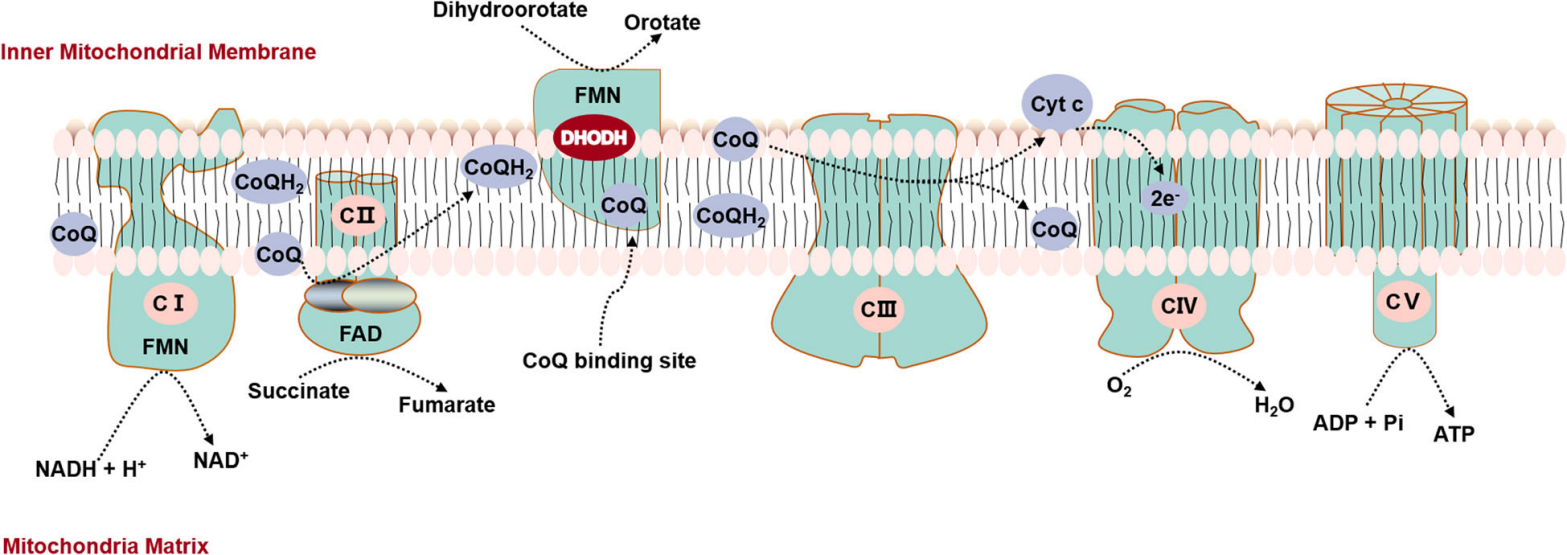
DHODH and mitochondrial respiratory chain. The mammalian mitochondrial electron transport chain comprises four enzyme complexes located in the mitochondrial inner membrane: complexes I, II, III, and IV. Complexes I and II transfer reducing equivalents from NADH and succinate to complex III via the ubiquinone pool, respectively, and complex III further transfers these equivalents to complex IV through cytochrome c. Electrons from complex IV are eventually transferred to dioxygen, subsequently producing water. ATP synthase generates ATPs by oxidative phosphorylation utilizing the transmembrane electrochemical gradient maintained by proton pumping activities of complexes I, III, and IV [36]. DHODH converts dihydroorotate to orotate, generating electrons that are transferred via redox-cycling of ubiquinone to complex III [2]. Thus, the de novo synthesis of pyrimidine nucleotides is coupled to the mitochondrial respiratory chain via DHODH [47]. Abbreviations: CI, complex I; CII, complex II; CIII, complex III; CIV, complex IV; CV, complex IV; FAD, flavin adenine dinucleotide; FMN, flavin mononucleotide. Q, coenzyme Q (CoQ), so known as ubiquinone; QH_2_, the hydroquinone (antioxidant) form of CoQ, also known as ubiquinol; UMP, uridine monophosphate

Mitochondria plays important roles in energy metabolism in eukaryotic cells by produce ATP via electron transport chain enzyme complexes (I-IV) which are located in the mitochondrial inner membrane [27]. Complexes I and II transfer reducing equivalents from NADH and succinate to complex III via the ubiquinone pool, respectively, and complex III further transfers these equivalents to complex IV through cytochrome c [36]. Electrons are eventually transferred to dioxygen, subsequently producing water [36]. ATP synthase generates ATPs by oxidative phosphorylation utilizing the transmembrane electrochemical gradient maintained by proton pumping activities of complexes I, III, and IV [36] (Fig. 2). The human DHODH protein resembles mitochondrial-targeting pre-sequences in its *N*-terminal segment and contains a bipartite signal that governs import and correct insertion into the mitochondrial inner membrane [2, 20]. As being located in mitochondria inner membrane, DHODH has inextricable link with mitochondria function.

DHODH is located between the complex II and complex III [4] (Fig. 2) and involved in electros production when it converts dihydroorotate to orotate [2]. Further, DHODH physically interacts with complexes II and III though the specific mechanism of this interaction awaits to be elucidated [22, 27]. Thus, inhibition of DHODH results in impairment of complex II and III function under hypoxia and nutrient-deprived conditions [27, 35, 48–50], followed by dysfunction of oxidative phosphorylation (OXPHOS) and aerobic glycolysis in activated T cells [51]. Meanwhile, inhibition of mitochondrial complex III also can suppress the function of DHODH by reducing turnover of ubiquinone which is essential for DHODH activity, thus reducing the pyrimidine de novo biosynthesis [52].

Mitochondria produce cellular energy by OXPHOS [53]. Essential protein subunits of OXPHOS complexes are assembled from both nuclear DNA (nDNA) and mitochondrial DNA (mtDNA) genes [53]. The reduction of pyrimidine nucleotide synthesis by inhibition of DHODH results in shortage of nDNA and mtDNA for OXPHOS complexes. Depletion of mtDNA induces mitochondria devoid of a functional respiratory chain, further decreases ATP production and leads to cell proliferation inhibition [54, 55]. For example, DHODH inhibition affects ATP depletion in breast cancer cells [56]. Conversely, these studies also revealed that DHODH knockout cells feature normal levels of OXPHOS-derived ATP and bioenergetics [57]. It is found that DHODH only contributed to no more than 5–10% of conventional (ATP-coupled) oxygen consumption in OXPHOS in 4T1 and B16 cells, suggesting that the direct contribution of DHODH to ATP generation may be negligible at baseline [7]. While the mechanism why DHODH inhibition makes ATP levels unchanged remains to be studied.

### ROS production and DHODH

Reactive oxygen species (ROS), which includes superoxide (O_2_∙), hydrogen peroxide (H_2_O_2_), and the hydroxyl radical (OH∙) are formed by the chemical reduction of O_2_ [58]. Mitochondria are a major source of ROS production within cells (mROS). There are at least 10 sites in the mitochondrial electron transport chain and matrix that are able to produce superoxide/H_2_O_2_ at measurable rates [48]. Cancer cells struggle to achieve a delicate redox balance to ensure survival [54]. Mounting evidence shows that mROS are critical for intracellular redox signaling by which they contribute to a plethora of cellular processes such as proliferation [56, 59]. ROS can induce DNA damage, activate oncogenes, block the function of tumor suppressors, and drive migratory signaling, which further results in tumorigenesis [56, 59]. On the other hand, ROS also can induce oxidation stress leading to cell apoptosis [54]. DHODH is a major contributor to mitochondrial oxygen consumption and ROS production in malignant tumor such as leukemia [47]. DHODH can generate superoxide and/or H_2_O_2_ directly at low rates and is capable of indirect production at higher rates from other sites through its ability to reduce the ubiquinone pool [14]. Conversely, recent studies prove that DHODH inhibition may also contribute to increased ROS. DHODH depletion partially inhibits the mitochondria complex III, decreases the mitochondrial membrane potential, and increases the generation of ROS [22]. Meanwhile, there is also a study that finds DHODH inhibitor-sensitive breast cancer cells reveal unchanged ROS production [56, 57]. Possible reasons are that DHODH induces ROS production which generally arises from other mitochondrial sites, and the function of these mitochondrial sites may affect the efficacy of ROS production under DHODH inhibition [14]. Moreover, the effect of DHODH in ROS level is relevant with some specific conditions [7]. For example, when cells are treated with anti-cancer agent fenretinide which can induce ROS production, DHODH suppression significantly reduces fenretinide-induced ROS generation [7, 55]. Taken together, the connection between DHODH and ROS in cancer is not fully understood and remains to be further verified.

## The various malignances and DHODH

**Fig. 3:**
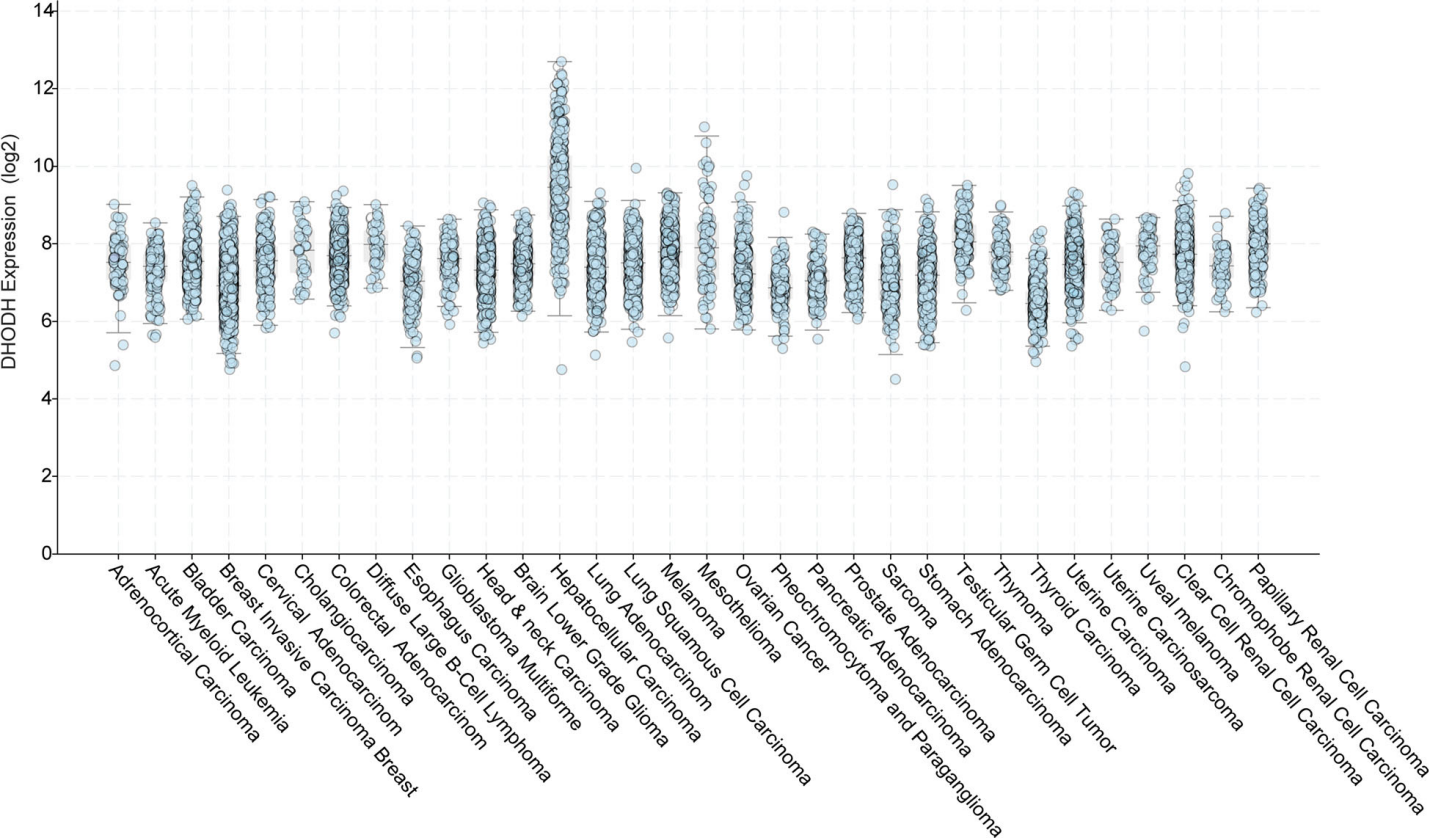
*DHODH* mRNA level in different malignancy types. The chart shows *DHODH* mRNA level in different kinds of malignancies. A circle represents a sample. All these data are concluded in *Cbioportal*, from TCGA Pan-Cancer Atlas Studies which cover 10967 samples from 32 studies

The overexpression of DHODH are observed in multiple malignance cells, including AML [60], skin cancer [61], colorectal cancer (CRC) [62], pancreatic cancer [63], breast cancer [56], lung cancer [64], multiple myeloma cells [65], neuroblastoma cells [48], renal cell carcinoma (RCC) [49], cervical cancer [50], and glioblastoma stem cells (GSCs) [25]. Especially, hepatocellular carcinoma exhibits the highest DHODH mRNA level among above malignance (Fig. 3). The overexpression of DHODH is associated with malignance growth and invasion.

### AML

AML, belonging to leukemia which ranks fifteen of 36 cancers in 185 countries in 2020 [66], is a clinically devastating disease with a quite variable prognosis and a high mortality rate [67]. Its 5-year overall survival is lesser than 50%, and in elderly patients only 20% will survive 2 years after diagnosis [67]. AML cells are found to be particularly sensitive to DHDOH depletion [68]. When in condition of pyrimidine starvation by DHODH inhibition, the AML blasts revealed cell death and differentiation with changes of morphology, cell surface marker expression, and gene expression [68]. Moreover, it is unraveled that inhibition of DHODH enables myeloid differentiation in human and mouse AML models via downregulating MYC which is a key transcription factor correlated with tumor cell differentiation [68, 69]. Besides, the differentiation effects by DHODH inhibition may include suppression of nucleic acid synthesis, cell cycle arrest, and influences in post-translational glycosylation of crucial proteins [39]. The mechanism of DHODH inhibition and myeloid differentiation still remains to be further manifested.

So far, two newly patented human DHODH inhibitors are currently being investigated for AML treatment: ASLAN003, currently being evaluated in Phase II clinical trial (clinical trial identifier: NCT03451084); BAY2402234, a compound by Bayer entered Phase I clinical trials in January 2018 (clinical trial identifier: NCT03404726) [60]. Meanwhile, isobavachalcone, a novel DHODH inhibitor can directly inhibit human DHODH and induce apoptosis and differentiation of AML cells [70]. PTC299, another novel DHODH inhibitor, has broad and potent activity against hematologic cancer cells in preclinical models [40]. DHODH inhibition also has strong effects in mixed-lineage leukemia gene MLL-fused leukemia cell lines [71]. Taken together, DHODH inhibitors promote differentiation of leukemia cells, decrease levels of leukemia-initiating cells, and improve survival in vivo, which demonstrates DHODH inhibition as a strategy for AML treatment [39].

### Breast cancer

Breast cancer, the fifth leading cause of cancer mortality worldwide, with 685,000 deaths [66], has now surpassed lung cancer as the leading cause of global cancer incidence in 2020, with an estimated 2.3 million new cases, representing 11.7% of all cancer cases [66]. DHODH is overexpressed in human breast cancer tissues (Fig. 3), and breast cancer cells expressing high DHODH show high sensitivity to DHODH inhibitors [56]. DHODH inhibitors lead to ATP depletion with maintained ROS level, S phase arrest and upregulate the expression of p53, p65, and STAT6 proteins in sensitive T-47D and MDA-MB-231 breast cancer cells, compared with non-sensitive MDA-MB-436 and W3.006 breast cancer cells [56]. PTEN-mutant human breast cancer cell lines display increased cell death overtime upon treatment with DHODH inhibitor leflunomide [29]. DHODH suppression induces sensitivity to TRAIL and replication stress in MCF-7 breast cancer cells [37, 72]. Furthermore, DHODH blockade in p53-mutant MDA-MB-231 and p53-deficient 4T1 breast cancer cells displays pyrimidine depletion on the cell cycle [37]. It reminds us of distinguishing the sensitivity of breast cancer cells to DHODH inhibitor by considering different molecular mechanism and DHODH inhibition as an alternative method for breast cancer treatment.

### CRC

CRC ranks third in terms of incidence of 6%, but second in terms of mortality of 5.8% in global cancer statics of 2020 [66] with 5-year relative survival ranges from greater than 90% in patients with stage I disease to slightly greater than 10% in patients with stage IV disease [73, 74]. DHODH shows significantly higher expression in CRC tumor tissue compared with normal samples using oncomine dataset analysis, which is consistent with a study that cell lines derived from the small and large intestine malignancies are most sensitive to DHODH knockdown [8]. As DHODH overexpression is associated with proliferation of CRC cells, inhibition of DHODH exhibited anti-proliferative effect against CRC cells. Moreover, the sensitivity of CRC cells to DHODH inhibitors are associated with the gene expression of cells. KRAS mutation is among the most prominent molecular markers in metastatic CRC patients, and mutations of KRAS render cells responsive to anti-cancer therapy such as anti-EGFR antibody treatment [73]. However, KRAS mutant CRC cells exhibit proliferation suppression in response to DHODH inhibitor brequinar (BRQ) treatment, which includes an increase in the steady-state concentrations of fructose 1,6-bisphosphate and change of metabolite levels including decreased glutamine and glutamate [63]. DHODH blockade and impairment of de novo pyrimidine biosynthesis by leflunomide trigger apoptosis in human CRC cells expressing transcriptionally active p53, which is consequential to the inhibition of the electron transport chain complex III [75]. When HCT116 CRC cells were treated with leflunomide, cell cycle arrest as well as increased switch to senescence is observed [75]. In addition, leflunomide effectively decreases proliferation of CRC cells via induction of replication and ribosomal stress in a p53- and checkpoint kinase 1 (Chk1)-dependent manner [37]. DHODH inhibition by leflunomide also strongly impairs CRC liver metastatic colonization compared with primary tumor growth [76]. HT29 cell lines are shown to be sensitized to TRAIL by BRQ [72]. Moreover, BRQ potentiates 5-fluorouracil antitumor activity in a murine model colon cancer by tissue-specific modulation of uridine nucleotide pools [77]. Similar results are found in MC38 colon carcinoma cells [56], which further display combined effect of DHODH inhibitors and 5-fluorouracil. Moreover, DHODH expression, with a predictive miR-502 binding site at position 245 to 251 in 3′-UTR of its mRNA, is directly regulated by miR-502 in CRC [62]. Ectopic expression of miR-502 precursor in human CRC cells HCT116 leads to significant decrease in DHODH mRNA and protein levels compared with negative miRNA transfection [62]. All these evidences prove that DHODH blockade could be a potential target in CRC treatment, especially in KRAS mutant, p53-deficient, or other genetic context CRC cells.

### Lung cancer

Lung cancer, the second most commonly diagnosed cancer and the leading cause of cancer death in 2020, approximately 11.4% in cancers diagnosed and 18.0% in deaths, is also the leading cause of cancer morbidity and mortality in men [66], whereas its incidence ranks third after breast cancer and CRC, and its mortality ranks second after breast cancer in women [66]. It mainly includes small cell lung cancer (SCLC) and non-small cell lung cancer (NSCLC) in terms of biological characteristics [78–80]. It is manifested that lung cancer cell lines show higher DHODH expression than normal lung cells and DHODH knockout exerts significant effects on lung cancer cell proliferation inhibition, suggesting that lung cancer cell lines are sensitive to DHODH inhibitors [8]. A recent study demonstrates that the sensitivity of SCLC cells toward disruption of the pyrimidine biosynthesis pathway enhanced, and DHODH inhibition by BRQ suppresses SCLC tumor growth and extends mice survival in vivo [72]. Downregulation of DHODH sensitizes SCLC cell U1690 to TRAIL-mediated apoptosis, and BRQ possesses significantly lower toxicity toward U1690 cells, which would be optimal for a combinatorial therapy [41, 69]. Besides, selective sensitivity to blockade of the de novo purine synthesis pathway is revealed in the variant subtype of SCLC [81], though the mechanism on how the variant subtype of SCLC exhibits sensitivity toward DHODH inhibition remains to be further elucidated [41]. It might show distinctions of sensitivity to DHODH inhibition in different gene context and tumors, which is an interesting direction to be further explored. Accordingly, under different genetic conditions in vivo, the decisive limitation of pyrimidine biosynthesis in tumors remains to be determined. Though related researches are not numerous, it seems to be a meaningful start for the future application of DHODH inhibitor in lung cancer therapy.

### Pancreatic cancer

Pancreatic cancer, with the occurrence of 2.6% and mortality of 4.7% [66], accounts for almost 466,000 deaths and 496,000 new cases because of its poor prognosis and is the seventh leading cause of cancer death in both sexes [66] with the overall average 5-year survival of 9% [74]. It is predicted that pancreatic cancer will surpass breast cancer as the third leading cause of cancer death by 2025 in 28 European countries [66]. DHODH reveals higher mRNA level in human pancreatic cancer tissues compared with normal pancreatic tissue [8]. A recent study reveals a greater increase in concentrations of newly synthesized UMP over time in human pancreatic ductal adenocarcinoma (PDAC) cell lines, suggesting that PDAC cells exhibit higher flux through the de novo pyrimidine synthesis pathway [41]. It manifests that DHODH may play a vital role in pancreatic cancer and its depletion might contribute to anti-cancer efficiency. Notably, DHODH inhibition induces cell cycle S phase arrest and mitochondrial depolarization in KP-4 human pancreatic cancer cells. In vivo preclinical studies demonstrate strong anti-cancer activity upon DHODH inhibition in a pancreatic tumor xenograft model [63]. Moreover, DHODH inhibitor teriflunomide not only shows in vitro anti-proliferative activity but also synergizes with the chemotherapeutic gemcitabine in pancreatic cancer cells, which is explained by interfering with PIM-3 kinase which is important in cell proliferation and protein synthesis of pancreatic cancer cells [82]. DHODH inhibition by Ter may exert its effect on PIM-3 and its downstream target leading to apoptosis [82]. Collectively, DHODH inhibitor may provide a novel way for pancreatic cancer treatment.

### Skin cancer

Skin cancers are one of the most common malignancies, particularly in the white population. The three commonest skin cancer types are basal cell carcinomas (BCC), squamous cell carcinomas (SCC) (also referred as nonmelanocytic skin cancer, NMSC) and cutaneous malignant melanoma (CM) (also designed as malignant melanoma of the skin or melanoma) [83]. Nonmelanoma skin cancer is responsible for over one million new cases of 6.2% and 64,000 deaths of 0.6% in 2020. While melanoma accounts for only 2% of all skin cancers, it causes most skin cancer deaths [84]. It is demonstrated that DHODH is mainly expressed in the epidermal hyperplasia, actinic keratoses (AK, the promotion phase), and Bowen’s disease, and invasive SCC specimens also display diffuse distribution [85]. Of note, DHODH activity is roughly twice higher in skin cancers than normal skin [85]. Recently, a study shows that DHODH, playing a critical role in UVB-induced energy metabolism reprogramming, is upregulated mainly resulting from transcriptional activation by signal transducer and activator of transcription 3 (STAT3) in a multistage model of UVB radiation-induced skin cancer. Importantly, inactivated DHODH or impaired electron transport chain blocks neoplastic transformation of keratinocytes [86]. DHODH blockade could be significant in the prevention or therapy of nonmelanoma skin cancers and possibly other hyperproliferative keratinocytic diseases [85]. Chronic inhibition of DHODH by leflunomide blocks UVB-induced cutaneous SCC initiation [87]. Moreover, it is found that BRQ and doxorubicin synergistically inhibited melanoma xenografts [88], and this combination significantly downregulated cell cycle regulatory proteins, cyclin B1, and upregulated its binding partner pcdc-2 and p21 [88]. It is demonstrated that DHODH inhibition abrogates the transcriptional elongation of genes, including Myc target genes and *mitfa* [89]. These genes are required for melanoma and multipotent neural crest cells which is an important precursor involved in melanoma pathogenesis proliferation [89]. Further, a DHODH inhibitor (R)-HZ00, which induced the accumulation of cells in S phase, an increase in p53 synthesis by inhibiting p53 degradation, and enhancement of the antitumor effect in human melanoma cell line ARN8, is uncovered [61]. Leflunomide abrogates the effective transcription elongation of genes required for neural crest development and melanoma growth in vivo, and nucleotide depletion reduces the chromatin occupancy of the RNA helicase protein DDX21 in human A375 melanoma cells [90]. DHODH inhibitor HZ-05 can suppress ARN8 melanoma cell growth and viability and induce induction DNA damage through activating p53-dependent transcription factor activity [35]. Given these observations found, DHODH inhibitor might be a bright method in the targeted therapy of melanoma.

### DHODH and other neoplastic diseases

DHODH inhibition can inhibit a variety of cancer cells, including multiple myeloma cells [65], neuroblastoma cells [48], RCC [49], cervical cancer [50], and GSCs [25].

Teriflunomide induces G1 cell cycle arrest via modulation of cyclin D2 and pRb expression and decreases phosphorylation of protein kinase B (PKB), p70S6K, and eukaryotic translation initiation factor 4E-binding protein-1 in multiple myeloma cells [65]. Leflunomide induces S phase cell cycle arrest and promotes cell apoptosis, further to suppress neuroblastoma cell proliferation in vitro and in vivo [48]. Leflunomide at high concentrations targets the canonical WNT/β-catenin signaling and induces the nucleocytoplasmic shuttling of β-catenin, subsequently promoting its proteasome-dependent proteolysis. Thus, DHODH inhibition can interrupt the canonical WNT/β-catenin signaling to induce RCC cell growth arrest and apoptosis [49]. Further, an fluorescence detection assay for measuring DHODH activity is developed in cultured HeLa cervical cancer cells, and in stage III stomach cancer and adjacent normal tissues from the same patient. Results indicate significantly DHODH shows higher activity in malignant tumor tissue than in adjacent normal tissue [50]. DHODH expression is also associated with increased grade and stage in glioma patients from the TCGA database [84]. Higher expression of pyrimidine synthesis genes including DHODH portends poor prognosis of patients with glioblastoma [25]. Targeting the pyrimidine synthetic rate-limiting step enzyme CAD or the critical downstream enzyme DHODH markedly inhibits GSC survival, self-renewal, and in vivo tumor initiation through the depletion of the pyrimidine nucleotide supply in rodent models [25].

Taken together, DHODH is releasing increasing potential abilities in various cancer therapies and it is worthy of expecting its more powerful utilization in other tumors.

## Co-expression network of DHODH

### Co-expressed genes with DHODH

**Fig. 4:**
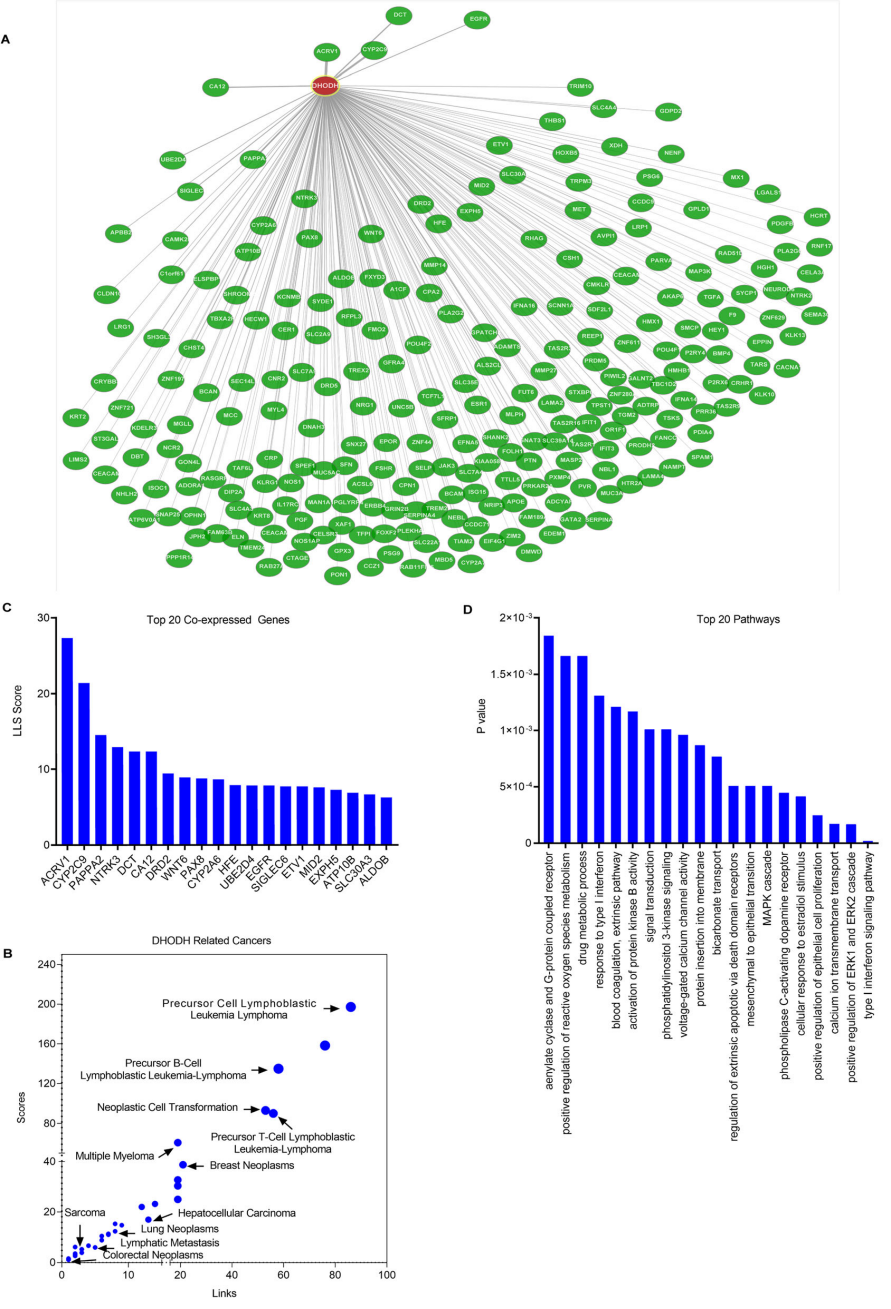
DHODH co-expressed genes. **a** Co-expression genes network with DHODH. **b** Relationship between DHODH co-expressed genes and different cancers. **c** The top 20 co-expressed genes with DHODH are ranked by sum of their LLS score. **d** Positively correlated top 20 pathways to DHODH co-expression genes by GeneSet analysis. Enriched terms by *p* value < 0.05. All these data are concluded from database *Coexpedia*. Abbreviations: ACRV1, acrosomal vesicle protein 1; CYP2C9, cytochrome P450 family 2 subfamily C member 9; PAPPA2, pappalysin 2; NTRK3, neurotrophic tyrosine kinase, receptor, type 3; DCT, dopachrome tautomerase; CA12, arbonic anhydrase XII; DRD2, dopamine receptor D2; WNT6, wingless-type MMTV integration site family member 6; PAX8, paired box 8; CYP2A6, cytochrome P450 family 2 subfamily A member 6; HFE, hemochromatosis; UBE2D4, ubiquitin conjugating enzyme E2D 4 (putative); EGFR, epidermal growth factor receptor; SIGLEC6, sialic acid binding Ig-like lectin 6; ETV1, ets variant 1; MID2, midline 2; EXPH5, exophilin 5; ATP10B, ATPase, class V, type 10B; SLC30A3, solute carrier family 30 (zinc transporter), member 3; ALDOB, aldolase, fructose-bisphosphate B

Various genes are found to be associated with DHODH through *Coexpedia*, which is a database widely applied for gene co-expression network analysis. Genes co-expressed with DHODH which were reported before are listed (Fig. 4a). Relationship among all these genes and different kinds of cancers are displayed (Fig. 4b). Lymphoma holds an important portion in these malignancies, which is in accord with the inhibitory effect of DHODH in T lymphocyte. Moreover, the top 20 co-expressed genes with DHODH are ranked according to the Lake Louise Score (LLS) score (Fig. 4c), including acrosomal vesicle protein 1 (ACRV1), cytochrome P450 family 2 subfamily C member 9 (CYP2C9), pappalysin 2 (PAPPA2), and neurotrophic tyrosine kinase, receptor, type 3 (NTRK3). GO analysis reveals the positively correlated top 20 pathways to DHODH co-expression genes (Fig. 4d), including type I interferon signaling pathway, positive regulation of ERK1 and ERK2 cascade, inflammatory response, positive regulation of extrinsic apoptotic signaling pathway via death domain receptors, MAPK cascade, positive regulation of phosphatidylinositol 3-kinase signaling, activation of protein kinase B activity, positive regulation of ROS metabolic process, drug metabolic process, and positive regulation of adenylate cyclase activity involved in G-protein coupled receptor signaling pathway.

### Co-expressed proteins with DHODH

**Fig. 5:**
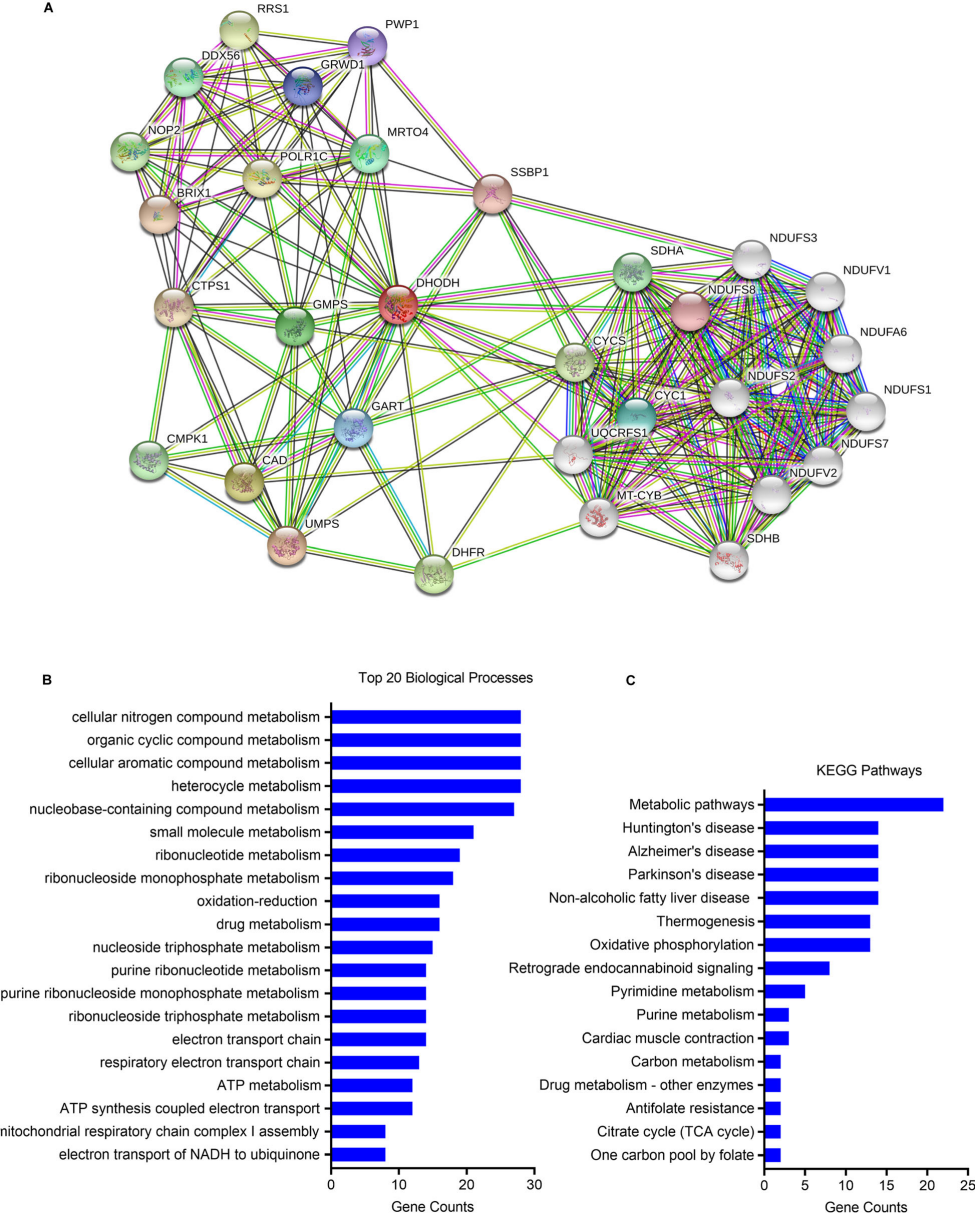
DHODH co-expressed proteins. **a** Co-expressed proteins with DHODH. **b** GO analysis concludes the top 20 biological process of proteins/genes co-expressed with DHODH. **c** KEGG analysis reveals the related pathways of proteins/genes co-expressed with DHODH. All these data are from STRING, which is a website used for protein-protein interaction network and functional enrichment analysis

A number of proteins which may be co-expressed with DHODH are found by STRING dataset. Here we mainly display the top 20 relative proteins with DHODH, as is depicted in Table 1 and Fig. 5.

**Table 1:** Co-expressed proteins with DHODH

Protein	Description
UMPS	Uridine 5′ monophosphate synthase; in the C-terminal section; belongs to the OMP decarboxylase family (480 aa)
CAD	CAD protein. This protein is a “fusion” protein encoding four enzymatic activities of the pyrimidine pathway (GATase, CPSase, ATCase, and DHOase); in the central section; belongs to the metallo-dependent hydrolases superfamily. DHOase family. CAD subfamily (2225 aa)
DHFR	Dihydrofolate reductase; key enzyme in folate metabolism. Contributes to the de novo mitochondrial thymidylate biosynthesis pathway. Catalyzes an essential reaction for de novo glycine and purine synthesis and for DNA precursor synthesis. Binds its own mRNA and that of DHFR2 (187 aa)
GMPS	GMP synthase [glutamine-hydrolyzing]; Involved in the de novo synthesis of guanine nucleotides which are not only essential for DNA and RNA synthesis, but also provide GTP, which is involved in a number of cellular processes important for cell division; Glutamine amidotransferase like class 1 domain containing (693 aa)
MRTO4	mRNA turnover protein 4 homolog; component of the ribosome assembly machinery. Nuclear paralog of the ribosomal protein P0, it binds pre-60S subunits at an early stage of assembly in the nucleolus and is replaced by P0 in cytoplasmic pre-60S subunits and mature 80S ribosomes (239 aa)
CYC1	Cytochrome c1, heme protein, mitochondrial. This is the heme-containing component of the cytochrome b-c1 complex, which accepts electrons from Rieske protein and transfers electrons to cytochrome c in the mitochondrial respiratory chain; Apoptosome (325 aa)
GART	Trifunctional purine biosynthetic protein adenosine-3; phosphoribosylglycinamide formyltransferase, phosphoribosylglycinamide synthetase, phosphoribosylaminoimidazole synthetase; in the central section; belongs to the AIR synthase family (1010 aa)
GRWD1	Glutamate-rich WD repeat-containing protein 1; histone-binding protein that regulates chromatin dynamics and minichromosome maintenance (MCM) loading at replication origins, possibly by promoting chromatin openness; WD repeat domain containing (446 aa)
PWP1	Periodic tryptophan protein 1 homolog; May play an important role in cell growth and/or transcription; Belongs to the WD repeat PWP1 family (501 aa)
NDUFS8	NADH dehydrogenase [ubiquinone] iron-sulfur protein 8, mitochondrial; core subunit of the mitochondrial membrane respiratory chain NADH dehydrogenase (Complex I) that is believed to belong to the minimal assembly required for catalysis. The immediate electron acceptor for the enzyme is believed to be ubiquinone (By similarity). May donate electrons to ubiquinone; NADH-ubiquinone oxidoreductase core subunits (210 aa)
SSBP1	Single-stranded DNA-binding protein, mitochondrial. This protein binds preferentially and cooperatively to ss-DNA. Probably involved in mitochondrial DNA replication. Associates with mitochondrial DNA (148 aa)
BRIX1	Ribosome biogenesis protein BRX1 homolog; Required for biogenesis of the 60S ribosomal subunit (353 aa)
CTPS1	CTP synthase 1. This enzyme is involved in the de novo synthesis of CTP, a precursor of DNA, RNA, and phospholipids. Catalyzes the ATP- dependent amination of UTP to CTP with either l-glutamine or ammonia as a source of nitrogen. This enzyme and its product, CTP, play a crucial role in the proliferation of activated lymphocytes and therefore in immunity; glutamine amidotransferase like class 1 domain containing (591 aa)
POLR1C	DNA-directed RNA polymerases I and III subunit RPAC1; DNA-dependent RNA polymerase catalyzes the transcription of DNA into RNA using the four ribonucleoside triphosphates as substrates. Common component of RNA polymerases I and III which synthesize ribosomal RNA precursors and small RNAs, such as 5S rRNA and tRNAs, respectively. RPAC1 is part of the Pol core element with the central large cleflunomidet and probably a clamp element that moves to open and close the cleflunomidet (By similarity) (346 aa)
RRS1	Ribosome biogenesis regulatory protein homolog; involved in ribosomal large subunit assembly. May regulate the localization of the 5S RNP/5S ribonucleoprotein particle to the nucleolus; belongs to the RRS1 family (365 aa)
CYCS	Cytochrome c; electron carrier protein. The oxidized form of the cytochrome c heme group can accept an electron from the heme group of the cytochrome c1 subunit of cytochrome reductase. Cytochrome c then transfers this electron to the cytochrome oxidase complex, the final protein carrier in the mitochondrial electron transport chain (105 aa)
NOP2	Probable 28S rRNA (cytosine(4447)-C(5))-methyltransferase; involved in ribosomal large subunit assembly. S-adenosyl-l-methionine-dependent methyltransferase that specifically methylates the C(5) position of cytosine 4447 in 28S rRNA (Probable). May play a role in the regulation of the cell cycle and the increased nucleolar activity that is associated with the cell proliferation (Probable); belongs to the class I-like SAM-binding methyltransferase superfamily. RsmB/NOP family (845 aa)
CMPK1	UMP-CMP kinase; catalyzes the phosphorylation of pyrimidine nucleoside monophosphates at the expense of ATP. Plays an important role in de novo pyrimidine nucleotide biosynthesis. Has preference for UMP and CMP as phosphate acceptors. Belongs to the adenylate kinase family. UMP-CMP kinase subfamily (228 aa)
SDHA	Succinate dehydrogenase [ubiquinone] flavoprotein subunit, mitochondrial; flavoprotein (FP) subunit of succinate dehydrogenase (SDH) that is involved in complex II of the mitochondrial electron transport chain and is responsible for transferring electrons from succinate to ubiquinone (coenzyme Q). Can act as a tumor suppressor; belongs to the FAD-dependent oxidoreductase 2 family. FRD/SDH subfamily (664 aa)
DDX56	Probable ATP-dependent RNA helicase DDX56; may play a role in later stages of the processing of the pre-ribosomal particle

## Mutation and alternation of DHODH

This section displays a series of charts that show the distribution of different types of mutations for DHODH.

**Fig. 6:**
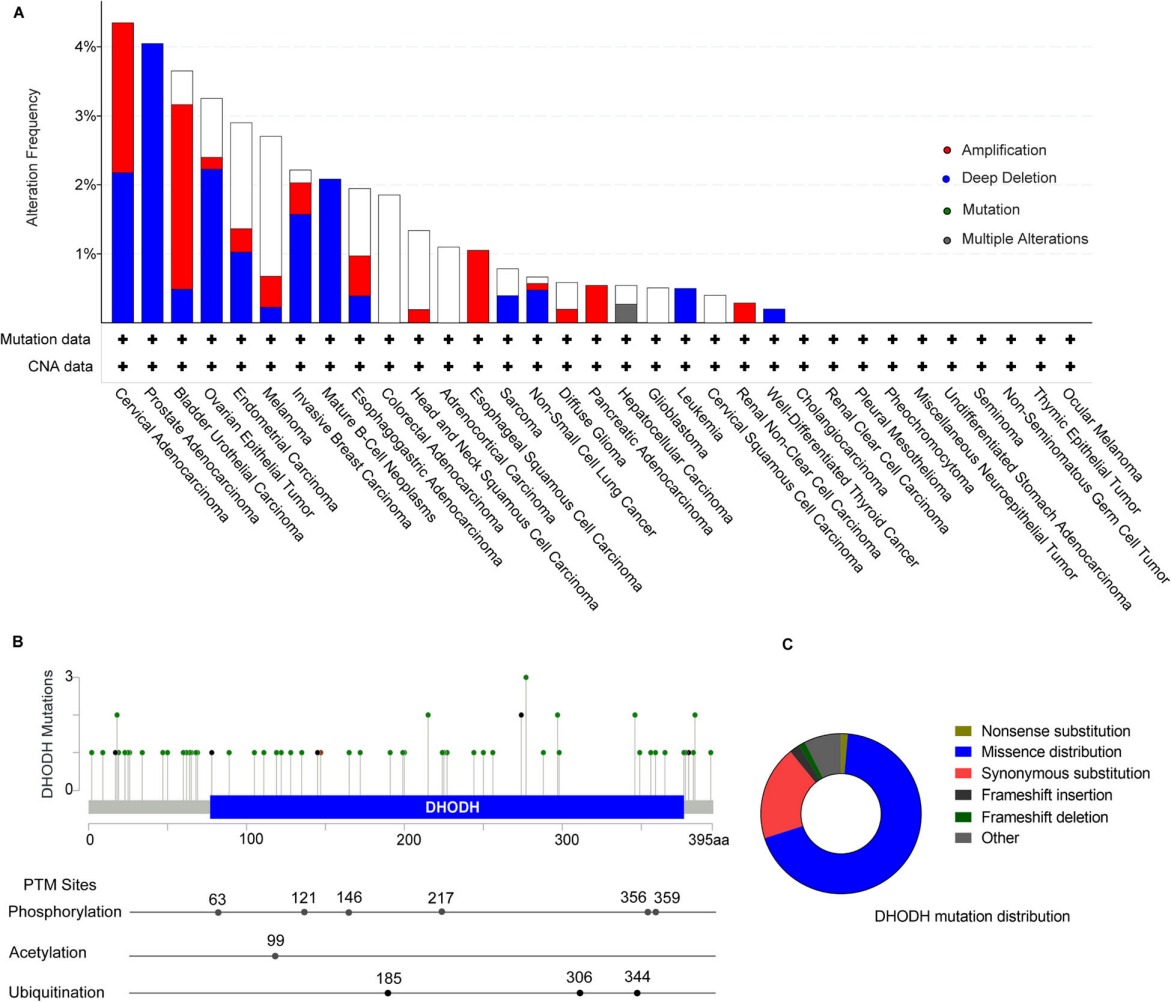
Mutation and alternation of DHODH. **a** All these data are from *Cbioportal.* **b** DHODH post-translational modifications (PTMs) sites, which are available for the Ensembl transcript ENST00000219240. Mutation diagram circles are colored with respect to the corresponding mutation types. In case of different mutation types at a single position, color of the circle is determined with respect to the most frequent mutation type. Mutation types and corresponding color codes are as follows: green, missense mutations; black, truncating mutations (nonsense, nonstop, frameshift deletion, frameshift insertion, splice site); brown, inframe mutations (inframe deletion, inframe insertion). All these data are from *Cbioportal.* **c** An overview of the types of DHODH mutation distribution. All these data are from *COSMIC*

Post-translational modification sites (PTMC) of DHODH protein are shown (Fig. 6b), which include phosphorylation at 63, 121, 146, 217, 356, and 359, acetylation at 99, and ubiquitination at 185, 306, and 344 [91–94]. In the total unique 217 samples, some verified DHDOH mutations are summarized, which includes nonsense substitution (0.92%), missense distribution (46.54%), synonymous substitution (12.90%), frameshift insertion (1.38%), frameshift deletion (0.92%), and other mutations (5.07%) (Fig. 6c).

## DHODH and non-neoplastic diseases

DHODH not only plays a vital part in malignancy therapy, but also in many other non-neoplastic diseases. DHODH inhibitors have been suggested for treating diseases such as adjuvant arthritis [95], lymphoproliferation and systemic lupus erythematosus [96], graft-versus-host disease [97], tubulointerstitial nephritis [98], proteoglycan-induced arthritis [99], glomerulonephritis [99], myasthenia gravis [100], diabetes [101], psoriasis, autoimmune diseases, plasmodium, bacterial infections [102], rheumatoid arthritis [103] and multiple sclerosis (MS) [51, 104, 105], Miller syndrome [106], edema [107], and hippocampal networks related epilepsy [108]. Moreover, DHODH inhibitors exhibited efficacy against virus, including respiratory syncytial virus [109], rotavirus [110], foot-and-mouth disease virus infection [111], flavivirus which contains dengue virus, Zika virus, and Japanese encephalitis virus [112], and notably, against RNA viruses including newly emerged coronavirus SARS-CoV-2 [113]. DHODH inhibitors are also found to be effective against oomycete phytophthora infestans [114], schistosomiasis [115, 116], invasive fungal infections [117], eumycetoma [118], and malaria [119–122].

In addition, DHODH, which mediates the de novo pyrimidine synthesis in actively proliferating T and B lymphocytes, also displays immunoregulation properties. Interference with immune cell proliferation represents a successful treatment strategy in T cell-mediated autoimmune diseases [51, 104]. DHODH inhibitor teriflunomide causes selective changes in T cell subset composition and T cell receptor repertoire diversity in patients with relapsing-remitting MS [51]. DHODH inhibition modulates T cell mitochondrial respiration with affinity-dependent effects in multiple sclerosis [104]. Further, human DHODH represents an important target for the treatment of hyperproliferative and inflammatory diseases, and its inhibition is beneficial to immunosuppressant and anti-proliferative effects in diseases such as RA. DHODH inhibitors BRQ and leflunomide show immunosuppressive and anti-proliferative activities, most pronounced in T cells [18]. Additionally, DHODH suppression might exert anti-inflammatory activities in autoimmune disease treatment by preventing the generation of proinflammatory Th1 effectors and promoting Th2 cell differentiation. The DHODH dysfunction directly inhibits NF-kB activity and disrupted cell migration, diminished cellular proliferation, and increased apoptosis, which contributes to Miller syndrome [22, 106].

A recent study displays that DHODH is a regulator of activity set points in hippocampal networks, and its inhibition can attenuate susceptibility to seizures. The DHODH inhibitor teriflunomide stably suppresses mean firing rates via synaptic and intrinsic excitability mechanisms by modulating mitochondrial Ca2^+^ buffering and spare respiratory capacity [108]. DHODH inhibition by teriflunomide is effective in suppressing FMDV infection [111]. Related DHODH inhibitors are currently in development now. DSM265, a triazolo-pyrimidine-based inhibitor of DHODH developed by Philips, is the first DHODH inhibitor able to reach clinical research for the treatment of malaria [123]. DHDOH point mutations, which are in the DSM265 binding site in *Plasmodium falciparum*, confer in vitro resistance to the clinical candidate DSM265 [71]. Significantly, DHODH blockade can be significantly realized in fighting against a broad spectrum of viruses especially in emerging serve coronavirus SARS-CoV-2, which may bring hope to the patients now suffering from severe COVID-19 and other infectious diseases caused by emerging and re-emerging viruses [113, 124]. It also widens the potential impact of DHODH inhibition on additional disease phenotypes. Diverse functions of DHODH uncover its value in multiple diseases not used exclusively for cancer.

## DHODH inhibitors

As for the importance of DHODH in tumorigenesis and metastasis, DHODH becomes an attractive target for anti-malignance drug development. Until now, many DHODH inhibitors with different structures have been reported [7], such as canonical DHODH inhibitors BRQ [125], leflunomide [2], teriflunomide [126], ALASN003 [127], BAY2202234 [43], and other novel inhibitors which show DHODH inhibition effects including viral growth inhibitory factor [128], PARP inhibitor [129], myeloid differentiation inducing agent [60], p53 activating factor [61], a natural product isobavachalcone [70], or vascular endothelial growth factor A (VEGF-A) mRNA translation inhibitor [40]. Over 90 patent applications involving DHODH inhibitors have been filed in the last decade [107, 130]. Notably, there is only one product, ASLAN003, that has been awarded orphan drug designation from the Food and Drug Administration (FDA) up to now [131].

As more and more novel DHODH inhibitors are emerging, it is significant to explore the binding modes between DHODH and DHODH inhibitors. Studies reveal that most DHODH inhibitors bind to the CoQ binding channel and display favorable activities [9]. For instance, BRQ bind to the CoQ binding site, and its biphenyl fragment forms a strong hydrophobic interaction with Leu46, Met43, Leu42, Leu68, and Met111, and the biphenyl fragment interacts with Tyr38 and Phe62 of DHODH [9].

Besides the binding modes between DHODH and DHODH inhibitors, some other factors also affect the efficiency of DHODH inhibitors like membrane lipid interactions [40, 132] and hypoxia conditions [36]. It is revealed that distinctive membrane-associated characteristic and mitochondrial location of DHODH may be associated with efficiency of DHODH inhibitors [133]. DHODH binds charged phospholipids which stabilize the flexible substrate- and drug-binding site to adhere to the mitochondrial membrane [40, 132]. The respiratory chain-coupled DHODH and its interplay with the surrounding lipids of the inner mitochondrial membrane provide a crucial direction for the development of DHODH-targeted inhibitors [133]. For example, the reason that DHODH inhibitor PTC299 displays potent activity is properly owing to the lipid bilayer of mitochondrial membrane facilitate the lipophilic PTC299 interacting with the DHODH binding pocket [40]. Thus, considering the impact of membrane lipid interactions on the drug-binding site may be another effective strategy to the design and development of novel DHODH inhibitors [132]. Furthermore, studies suggest the favorable targeting to DHODH in cancer cells living under hypoxia; it is worthwhile to dig into whether DHODH inhibitors with distinct scaffold also exhibit hypoxia-selective anti-cancer activity [36]. Taken together, all of these provide feasible methods for development of DHODH inhibitors (Table 2).

**Table 2:** DHODH inhibitors currently evaluated in clinical trials

Drug	Clinical trial	Disease	Clinical trial identifier	Study start date
Brequinar	Phase I/II	Acute myeloid leukemia	NCT03760666	November 26, 2018
Leflunomide	Phase I/II	Multiple myeloma	NCT02509052	November 2, 2015
Teriflunomide	Phase IV	Multiple sclerosis	NCT01970410	October 2013
ASLAN003	Phase II	Acute myeloid leukemia	NCT03451084	January 5, 2018
BAY2402234	Phase I	Leukemia	NCT03404726	March 29, 2018
IMU-838	Phase II	Ulcerative colitis	NCT03341962	March 15, 2018
PTC299	Phase I	Acute myeloid leukemia	NCT03761069	October 29,2018

All the information above originated from *ClinicalTrials.gov* (https://clinicaltrials.gov/). In note, this table only refers to currently ongoing clinical trials in which DHODH inhibitors are applied alone, but do not cover previous failed or completed clinical trials.

### Conventional DHODH inhibitors

#### BRQ

BRQ is originally developed for the therapy of organ transplant rejection, and the preclinical and clinical development of BRQ as an anti-cancer agent in 1997 [9, 18, 134]. Earlier investigation has revealed that BRQ is a powerful inhibitor predominantly for the dehydrogenase and not for the oxidase activity [135], and then BRQ is discovered to be one of the strongest known inhibitors of human DHODH with an IC_50_ value of 1.8 nM [125, 136]. Meanwhile, it is shown that both BRQ and its analog are located in the DHODH proposed ubiquinone binding channel [18]. BRQ can compete with ubiquinone and its core part, 2-phenyl 5-quinoline carboxylic acid (PQC), which also has a competitive inhibitory effect on ubiquinone [126]. BRQ structural analog not only interacts with DHODH through a carboxylic acid group, but also binds extensively to serum albumin, which alters their free unbound plasma concentration [61]. The in vivo study shows BRQ activates a p53-dependent reporter in cells at low concentrations and inhibits tumor growth. BRQ exhibits strong in vivo antitumor activity through DHODH depletion in a KRAS mutant pancreatic xenograft model and induces a decrease in steady-state glutamate concentration [63]. It also significantly sensitizes U1690 cells to TRAIL-mediated apoptosis [72].

BRQ is evaluated in the Phase I and II trials of patients with advanced solid tumor malignancies [107], such as squamous cell carcinoma of the head and neck [137], lung cancer [64], gastrointestinal cancer [138], and breast cancer and melanoma [139, 140]. However, it does not get different Phase II clinical trials where it demonstrated only very modest anti-cancer effects on various solid tumors. Moreover, its narrow therapeutic window further hinders clinical application [141]. Increased toxicity of BRQ and its analogs is revealed when given in combination with cyclosporine. The side effects observed include leukocytopenia, thrombocytopenia, reduced body weight gain or body weight loss, thymic atrophy, cellular depletion of bone marrow and splenic white pulp, and villous atrophy in jejunum [134]. In 2018, BRQ has been reapplied in clinical trials of AML, which reveals that BRQ may have potential in malignancy therapy.

#### Leflunomide

Leflunomide (HR486) is an isoxazole derivative that originated from an anti-inflammatory drug development program at Hoechst during the 1980s in order to control autoimmune diseases [30, 35]. Leflunomide binds with DHODH within the tunnel which can directly lead to the bound FMN and provide access to ubiquinone so as to block ubiquinone to the active site [2]. It exhibits both anti-inflammatory and immunomodulatory properties with a different structure from other known immunomodulatory drugs [142]. The anti-inflammatory effects of leflunomide are associated with inhibited de novo synthesis of pyrimidine nucleotides (and consequently DNA and RNA), inhibited oxidative damage, and reduced proinflammatory cytokine expression in immune response cells [143, 144]. Leflunomide exerts effects on murine models of lymphoproliferation and systemic lupus erythematosus [96], graft-versus-host disease [97], proteoglycan-induced arthritis [99], and diabetes [101], and on rat models of adjuvant arthritis [95], tubulointerstitial nephritis [98], glomerulonephritis due to anti-basement membrane antibody [99], and the anti-acetylcholine receptor-induced model of myasthenia gravis [100]. In 1999, leflunomide was approved by the FDA and first used as an oral disease-modifying antirheumatic drug (DMARD) for the treatment of active RA refractive to methotrexate.

In addition to above efficiency, leflunomide shows antitumor potency against various malignancies. DHODH inhibition by leflunomide induces upregulation of p53 in Hela cells [52], inhibits proliferation of PTEN-mutant human breast cancer cell lines [29, 145], and contributes to RCC S phase arrest and autophagy [49]. Leflunomide induces S phase arrest and accumulation of cyclin E in breast cancer cells, which is further supported in DHODH-silenced cells [37]. DHODH suppression by leflunomide decreased cell motility in breast cancer cells, which could explain the anti-metastatic effect of leflunomide [37]. Leflunomide-treated mice show decreased DHODH activity and fail to develop pre-malignant and malignant lesions [86]. DHODH inhibition by leflunomide also leads to an almost complete abrogation of neural crest development in zebrafish and to a reduction in the self-renewal of mammalian neural crest stem cells [89]. However, severe side effects of leflunomide are observed including diarrhea, abnormal liver functions, hypertension, myelosuppression, nausea, and hair loss [107] during its administration. In 2010, the FDA gave leflunomide a black box warning due to its causing of acute liver failure in humans though the underlying mechanism of its toxicity remains unclear. The severe side effects hinder its further clinical use.

#### Teriflunomide

In 1979, teriflunomide (A77-1726, Ter), the active metabolite of leflunomide, was originally found as a drug for rheumatoid arthritis and multiple sclerosis treatment. Leflunomide is rapidly converted into its active metabolite teriflunomide via a non-enzymatic, base-caused isoxazole ring-opening reaction in the plasma and intestinal mucosa [146]. Teriflunomide is a non-competitive inhibitor with ubiquinone with an IC_50_ value of approximately 600 nM against DHODH activity [143]. Compared with leflunomide, teriflunomide is well tolerated, has a long half-life of more than 2 weeks, and can be delivered into the brain, which supports that teriflunomide is a particularly attractive choice of pyrimidine-targeting strategy [36, 38].

Teriflunomide exhibits antitumor effects by inhibiting DHODH activity in different malignances. It significantly reduces proliferation of primary microglia [147], GSCs [25], etc. Teriflunomide treatment of GSCs with either EGFR amplification or PTEN deletion inhibits pyrimidine biosynthesis and cells proliferation. Moreover, teriflunomide displays combinatorial function with either lapatinib or BKM120 in reduction of pyrimidine synthesis [25]. The in vivo mechanism studies have demonstrated that teriflunomide can inhibit adhesion molecules, cytokines, protein tyrosine kinases, nuclear factor-kB (NF-kB) activation, and cyclooxygenase 2 activity, suggesting that teriflunomide in addition to its anti-proliferative effects may also impact signal transduction, migration, and inflammatory processes [147–149]. Besides inhibiting malignancies, teriflunomide contributes to apoptosis in normal and transformed human keratinocytes [85], suggesting its potential toxicity against normal tissues. The common adverse events including headache, nausea, diarrhea, fatigue, elevated alanine aminotransferase levels, hair thinning or decreased hair density, and urinary tract infection are observed in its clinical use [107, 150, 151].

#### BAY2402234

BAY2402234 is a novel, potent, selective, and orally bioavailable DHODH inhibitor that shows monotherapy efficacy and differentiation induction across multiple AML subtypes [43]. It is developed by Bayer and has entered Phase I clinical trials in January 2018 (Clinical trial identifier: NCT03404726). In May 2018, the patent containing the BAY2402234 structure and a description of the activity profile has been published [60]. BAY2402234, binding the ubiquinone binding site of DHODH between the N-terminal helices, can concentration-dependently inhibit human full-length DHODH enzyme with an IC_50_ value of 1.2 nM [43]. Further, BAY 2402234 has moderate plasma protein binding, low blood clearance, and a high volume of distribution [43]. Additionally, BAY2402234 can downregulate key enzymes in UDP GlcNAc metabolism Glucosamine-Phosphate N-Acetyltransferase 1 and UDP-N-Acetylglucosamine Pyrophosphorylase 1, which may account for previous observation of global decreases in protein N-acetyl glycosylation after DHODH inhibition [39, 43]. As so far, the researches related to BAY2402234 are not abundant for its new discovery in 2018. It is shown that BAY2402234 induces differentiation and inhibits proliferation in AML cell lines across multiple AM subtypes by inhibiting DHODH, including ten leukemia cell lines representing diverse AML subtypes, with a low range of IC_50_ [43]. Significantly, BAY2402234 induces profound transcriptional changes, prolongs survival and induces differentiation in AML PDX models [43]. However, the side effects and anti-proliferation effects in other tumors remain to be explored.

#### ASLAN003

ASLAN003 (LAS186323), a bioavailable and novel DHODH inhibitor, was first discovered by Almirall, S.A., and global rights to the compound have been granted to ASLAN Pharmaceuticals Singapore in 2012. It is currently being evaluated in Phase II clinical trial (clinical trial identifier: NCT03451084) in AML patients [60, 127]. ASLAN003 is an effective inhibitor of human DHODH with an IC_50_ value of 35 nM. It can induce differentiation of AML cells in vitro and in vivo and trigger apoptosis, inhibit protein synthesis, and activate AP-1 transcription. Meanwhile, gene expression of four members of eukaryotic translation initiation factor (eIF) family, EEF1B2, EIF4B, EIF3L, EEF1B2P3, were significantly decreased in ASLAN003-treated cells. It has been proven tolerable in the first phase of single and multiple escalating dose clinical trials in healthy volunteers [127], while its side effects and antitumor functions in other cancers need to be further manifested.

### Other novel DHODH inhibitors

In recent years, novel DHODH inhibitors are massively emerging, which include analogs of BRQ [60, 102], IMU-838, and its active metabolite vidofludimus [152–154], PTC299 [40, 155], DD264 [128], plant extract Celastrol [156], protein kinase inhibitors OSU-03012 and TAK-632 [112, 130, 157], isobavachalcone [70], tetrahydroindazole (HZ) analogs [35, 61], alkaloid cerpegin-related P1788 [158], compound 11 [79], etc. (Table 3).

**Table 3:** Molecular structures and IC_50_ values for recently developed novel DHODH inhibitors

Compound	Structure	Disease	Activity/IC_**50**_	Reference
C07		AML	1.3 μM	[107]
DD264		Measles virus	15 μM	[107, 128]
DSM265		Malaria	41 μg/mL	[123]
DSM430		Malaria	3.7 μg/mL	[123]
DSM450		Malaria	Higher than 43 μg/mL	[123]
GSK983		Chronic myelogenous leukemia K562 cells	21 nM	[159]
HZ-05		Melanoma	32 ± 2 nM	[35, 61]
IBC		AML Hl60 cells	0.36 ± 0.05 μM	[70]
ML390		AML	0.56 μM	[107]
IMU-838		Ulcerative colitis, multiple sclerosis	/	[153, 160]
OSU-03012		/	/	[112, 130]
PP-001		Inflammation and chorioretinal neovascularization	Lower than 4 nM	[107, 161]
P1788		/	EC_50_ 44 μM	[158]
TAK-632		/	/	[112, 130]
Vidofludimus (4SC-101)		Colitis, systemic lupus erythematosus	0.134 ± 0.016 μM	[153]
Compound 1		/	6 nM	[162]
Compound 2		/	773 μM	[162]
Compound 3		Rheumatoid arthritis	280 nM	[18]
Compound 4		Rheumatoid arthritis	2 nM	[18]
Compound 5		/	K_i_ 2.8 μM	[163]
Compound 6		/	15 nM	[102]
Compound 7		/	81 nM	[102]
Compound 8		/	69 nM	[102]
Compound 11l		AML CRC Melanoma	4.5 nM	[164]
Compound 11k		AML CRC Melanoma	9.0 nM	[164]

Benzimidazole derivatives have shown a wide variety of pharmacological activities including dual PARP and DHODH inhibition. It is considerable to further study the binding mechanisms and enhance their potential as inhibitors of PARP-1 and DHODH [129]. IMU-838, a novel DHODH inhibitor, acts on activated T and B cells without affecting other immune cells, which allows the immune system to keep functioning [160]. Vidofludimus, the active moiety and free acid form of IMU-838, is a potent and selective inhibitor DHODH [152–154]. It can inhibit the intracellular metabolism of activated immune cells by blocking DHODH and inhibit colonic interleukin-17 and improve colitis in rats [154]. Lucas-Hourani et al. find a novel DHODH inhibitor DD264 with dual effects of immune-stimulation and antiviral through screening 41353 compounds. However, the antiviral property of pyrimidine biosynthesis inhibitors is not a direct consequence of pyrimidine deprivation on the virus machinery, but rather involves the induction of cellular immune response [128]. Antiviral activity of DD264 is found strictly dependent on cellular gene transcription and nuclear export machinery and required interferon regulatory transcription factor 1 (IRF1) transcription factor. Celastrol, extracted from “Thunder of God Vine,” is a DHODH inhibitor and promising anti-cancer natural product. Celastrol could induce apoptosis of acute promyelocytic leukemia (APL) cells via p53-activated mitochondrial pathway [156]. Recently, Abt et al. substantiate that two protein kinase inhibitors OSU-03012 and TAK-632 found by metabolic modifier screen, competing with ubiquinone, engage in DHODH through crystallography studies. Notably, OSU-03012 and TAK-632 bind in the same hydrophobic tunnel of DHODH as known inhibitors BRQ and teriflunomide. By competitively inhibiting the binding of ubiquinone, these compounds prevent DHODH from completing its redox cycle and effectively abrogate its activity [130]. Meanwhile, OSU-03012 and analogs can inhibit pyrimidine biosynthesis in host cells to inhibit viral replication, specifically illustrating regulation of DHODH activity [112, 130]. Isobavachalcone is a novel DHODH inhibitor which derived from traditional Chinese medicine psoralea corylifolia. It directly inhibits human DHODH and triggers apoptosis and differentiation of AML cells. Oral administration of isobavachalcone suppresses subcutaneous HL60 xenograft tumor growth without obvious toxicity. Importantly, combining isobavachalcone with adriamycin prolong mice survival in an intravenous HL60 leukemia model [70]. Ladds et al. identified over 100 small molecules activating p53 in cells and found active enantiomer (R)-HZ00 can inhibit DHODH through target deconvolution. Twelve other DHODH inhibitor chemotypes are detailed among the p53 activators, which identifies DHODH as a frequent target for structurally diverse compounds [61]. Recently, several HZ analogs are further to be optimized and display anti-proliferation effect on ARN8 melanoma cells [35]. PTC299 is a novel, orally bioavailable small molecule that selectively inhibits VEGF receptor protein synthesis at the post-transcriptional level. Recent study demonstrated that PTC299 is a more potent inhibitor of DHODH in isolated mitochondria. PTC299 has advantages over previously reported DHODH inhibitors, including greater potency, good oral bioavailability, and lack of off-target kinase inhibition and myelosuppression. PTC299 is currently in Phase I clinical trial against AML patients (clinical trial identifier: NCT03761069) and thus may be useful for the targeted treatment of hematologic malignancies [40]. 10580, a novel DHODH inhibitor, found through high-throughput drug screening, decreases pyrimidine nucleotide levels and enhances sex-determining region Y–box 2 nuclear export by antagonizing the enzyme activity of DHODH [165]. P1788, a compound chemically related to the alkaloid cerpegin, is a new class of DHDOH inhibitors. Pyrimidine depletion by P1788 amplifies cellular response to both type I and type II interferons and induces DNA damage [158]. Compound 11l and 11k, newly found in 2020, can significantly induce ROS production and mitochondrial dysfunction and inhibit leukemia cells and solid tumor cell proliferation [164].

## Prospects of DHODH inhibitors in cancer therapy

DHODH is a promising target in multiple malignancy therapies. When applying in specific genetic context, DHODH inhibitors might exert more targeted and meaningful effects on malignancy therapy. Recent studies have illustrated that DHODH inhibitors are more effective against malignancies with specific genetic context, such as melanomas carrying BRAF (V600E) mutation [89] and glioblastoma stem cells [25] and breast cancer with PTEN deletion [25, 29] and pancreatic cancer with KRAS mutation [63]. Therefore, the specific genetic context for DHODH inhibitor treatment need to be further explored.

Meanwhile, combination of DHODH inhibitors with other anti-cancer drugs is a promising approach to achieving clinical benefits in various tumor therapies as DHODH inhibitors alone have limited therapeutic windows and may not achieve desired outcomes. Studies verify that combination of DHODH inhibitors with traditional cell toxicity anti-cancer drugs synergistically inhibits tumor proliferation. Gemcitabine, a first-line choice for pancreatic cancer, is an ideal combination partner for DHODH inhibitors [63]. As gemcitabine treatment leads to increased de novo pyrimidine synthesis in pancreatic cancer cells, combination of DHODH inhibitor Ter with gemcitabine reversed gemcitabine-induced nucleoside synthesis and thus exhibits synergistic effect against pancreatic cancer proliferation [82]. Furthermore, teriflunomide combined with the genotoxic agents such as 5-fluorouracil (5-FU) [87], melphalan, treosulfan, doxorubicin, dexamethasone, and bortezomib reveal the synergistic and additive activity in multiple myeloma [65] and UVB-induced cSCC [87] proliferation suppression. Combination treatment of BRQ and doxorubicin results in significant anti-proliferation effects on melanoma cells both in vitro and in vivo [88].

Besides traditional cell toxicity of anti-cancer drugs, DHODH inhibitors in combination with novel targeted anti-cancer drugs attracted much attention. Combination of DHODH inhibitors with mitochondria complex I inhibitors can interfere two crucial steps of pyrimidine biosynthesis, which may provide a broad-spectrum and effective antitumor strategy [57]. Combination of leflunomide and the Chk1 inhibitor reduces proliferation and induces massive cell apoptosis of p53-deficient breast tumors compared with leflunomide treatment alone [37]. Given that p53 is generally mutated in more than 50% of tumors, the simultaneous inactivation of DHODH and Chk1 kinase is a potential method for the treatment of refractory p53 mutant tumors [37]. As salvage and de novo pyrimidine pathways are suppressed by ATR blockade [157], combination of ATR inhibitor and OSU-03012, a DHODH inhibitor, reveals synergy against pancreatic ductal adenocarcinoma [130]. These observations suggested that DHODH inhibitors may have utility in conjunction with DNA damage response pathway inhibitors [130, 157]. Meanwhile, in case of DHODH inhibitors ineffective due to efficient uridine salvage synthesis in certain cells, combination DHODH inhibitors with non-competitive pyrimidine salvage enzyme uridine-cytidine kinase 2 (UCK2) inhibitors was able to suppress nucleoside synthesis and inhibit cell proliferation [166]. Deoxycytidylate deaminase, converting deoxycytidine monophosphate (dCMP) to dUMP, is an enzyme that supplies the vast majority of dUMP for cancer cells [41, 167], and cells with high expression of DCTD are better able to maintain dUMP pools when the de novo pyrimidine synthesis pathway is inhibited, suggesting that combination of DHODH and DCTD inhibitors maybe a significant antitumor strategy, though the utility requires further investigation [41, 168, 169].

Although combined targeting strategies offer powerful methods for cancer therapy, the limitations are still existent, including alternate concentrations of inhibitors which are required to achieve therapeutic doses in order to satisfy distribution in different organs in the clinical trial. Combination BQR with other immuno-suppression inhibitors shows altered pharmacokinetics, including increased drug toxicity and decreased drug efficacious doses, suggesting pharmacokinetics and the therapeutic window in combination treatment may be a significant focus [134]. Taken together, DHODH inhibitors and anti-cancer drug combination may be a promising method of cancer therapy.

## Conclusions

DHODH, a key enzyme in the de novo pyrimidine synthesis pathway, plays a crucial role in tumorigenesis via influencing pyrimidine metabolism and mitochondria function. Inhibiting DHODH has gradually become a hit spot in malignancy therapy. Novel DHODH inhibitors are springing up at an accelerated pace in recent decades. Significantly, combined target therapy seems to be a particularly promising strategy, especially under specific genetic contexts, due to the re-sensitive of tumors to traditional chemotherapy. Meanwhile, the highly specific DHODH inhibitors and more appropriate administration dosage may improve efficacy and adverse side effects associated with traditional DHODH treatment, which is required to further investigate for the development of DHODH-targeted inhibitors in the near future. Collectively, DHODH, revealing multiple biological functions in neoplastic and non-neoplastic diseases, has broad-spectrum prospective remained to be explored.

## Data Availability

Not applicable.

## Abbreviations

5-FU: 5-Fluorouracil
AK: Actinic keratoses
ALDOB: Aldolase, fructose-bisphosphate B
AML: Acute myeloid leukemia
ACRV1: Acrosomal vesicle protein 1
ATR: Ataxia telangiectasia and Rad3-related kinase
ATP10B: ATPase, class V, type 10B
APPA2: Pappalysin 2
BCC: Basal cell carcinomas
CA12: Arbonic anhydrase XII
CAD: Carbamoylphosphate synthetase II (CPSII), aspartate transcarbamoylase (ATCase), dihydroorotase (DHOase)
CHK1: Checkpoint kinase 1
CM: Cutaneous malignant melanoma
CoQ: Coenzyme Q
COVID-19: Coronavirus disease 2019
cSCCs: Cutaneous squamous cell carcinomas
CTP: Cytidine triphosphate
CYP2A6: Cytochrome P450 family 2 subfamily A member 6
CYP2C9: Cytochrome P450 family 2 subfamily C member 9
dCMP: Deoxycytidine monophosphate
DCTD: Deoxycytidylate deaminase
DCT: Dopachrome tautomerase
dCTP: Deoxycytidine triphosphate
DHODH: Dihyrdroorotate dehydrogenase
DHO: Dihydroorotate
dUDP: Deoxyuridine diphosphate
DRD2: Dopamine receptor D2
dUMP: Deoxyuridine monophosphate
EGFR: Epidermal growth factor receptor
eIF: Eukaryotic translation initiation factor
ER: Endoplasmic reticulum
ETC: Electron transport chain
ETV1: ets variant 1
EXPH5: Exophilin 5
FMN: Flavin mononucleotide
GATA-2: GATA-binding factor 2
GlcNAc: N-acetylglucosaminylation
Gln: Glutamine
GSCs: Glioblastoma stem cells
HFE: Hemochromatosis
HIF1: Hypoxia-inducible factor 1
HZ: Tetrahydroindazoles
IBC: Isobavachalcone
IRF: Interferon regulatory transcription factor 1
KRAS: Kirsten rat sarcoma viral oncogene
LLS: Lake Louise Score
MLL: Mixed-lineage leukemia gene
MID2: Midline 2
mROS: Mitochondria reactive oxygen species
mtDNA: Mitochondrial DNA
NAD^+^: Oxidized nicotinamide adenine dinucleotide
NADH: Reduced nicotinamide adenine dinucleotide
nDNA: Nuclear DNA
NMSC: Nonmelanocytic skin cancer
NSCLC: Non-small cell lung cancer
NTRK3: Neurotrophic tyrosine kinase, receptor, type 3
ODC: Orotidine-5'-decarboxylase
O-GlcNAc: O-linked N-acetylglucosaminylation
OMP: Orotidine 5'-monophosphate
OPRT: Orotate phosphoribosyltransferase
ORF: Open reading frame
OXPHOS: Oxidative phosphorylation
PAX8: Paired box 8
PDAC: Pancreatic ductal adenocarcinoma
PIMs: The PIM protein serine-threonine kinase family
PKB: Phosphorylation of protein kinase B
PLK: Polo-like kinase
PTEN: Phosphatase and tensinhomolog
POU3F2: POU domain, class 3, transcription factor 2
RB: Retinoblastoma protein
RCC: Renal cell carcinoma
SARS-CoV-2: Severe acute respiratory syndrome coronavirus 2
SCCs: Squamous cell carcinomas
SCLC: Small cell lung cancer
SIGLEC6: Sialic acid binding Ig-like lectin 6
SLC30A3: Solute carrier family 30 (zinc transporter), member 3
STAT3: Signal transducer and activator of transcription 3
TFMA: Mitochondrial transcription factor A
TM: Transmembrane domain
TRAIL: Tumor necrosis factor-related apoptosis-inducing ligand
UBE2D4: Ubiquitin conjugating enzyme E2D 4 (putative)
UCK2: Uridine-cytidine kinase 2
UDP: Uridine diphosphate
UMP: Uridine monophosphate
UMPS: Uridine monophosphate synthase
UTP: Uridine triphosphate
WNT6: Wingless-type MMTV integration site family member 6
